# Clinical features, genetic analysis, and efficacy evaluation of hematopoietic stem cell transplantation in a case of osteopetrosis caused by compound heterozygous variations in the *TNFRSF11A* gene: a case study

**DOI:** 10.3325/cmj.2026.67.315

**Published:** 2026-08

**Authors:** Xiaoyun Dong, Jiping Lv, Hui Dong, Linfei Li, Xuan Zheng, Zongyuan Liu

**Affiliations:** 1 Department of Child Health Care, Children's Hospital Affiliated to Zhengzhou University, Zhengzhou, China; ²Neonatal Intensive Care Unit, The Third Affiliated Hospital of Zhengzhou University, Zhengzhou, China; ³Institute of Pediatrics, Children's Hospital Affiliated to Zhengzhou University, Zhengzhou, China

## Abstract

Osteopetrosis is an uncommon inherited skeletal condition arising from impaired osteoclast activity, with its autosomal recessive forms being linked to mutations in the *TNFRSF11A* gene. This study reports on a three-year-old Chinese girl presenting with recurrent fractures, short stature, and macrocephaly. Radiological examinations revealed diffusely elevated bone mineral density and constricted medullary spaces. Meanwhile, whole-exome sequencing detected compound heterozygous alterations in the *TNFRSF11A* gene: c.1567 + 2T>A inherited from the father and c.630C>G from the mother. The child underwent haploidentical hematopoietic stem cell transplantation (HSCT) from the father. Post-transplant follow-up showed improved growth (height/weight), resolved anemia, and alleviated optic foramen stenosis. The patient remained free of hepatosplenomegaly. This case report suggests that HSCT holds therapeutic potential for *TNFRSF11A*-related osteopetrosis and highlights the clinical value of early genetic diagnosis and timely transplantation for optimizing patient prognosis. Larger studies are required to confirm these findings and further guide the clinical management of similar rare disease cases.

Osteopetrosis is an uncommon genetic skeletal condition resulting from disrupted physiological bone remodeling, which in turn impairs osteoclast function. Affected individuals typically exhibit elevated bone density, absent medullary spaces, stunted growth, an enlarged cranium, as well as progressive hearing and vision loss, enlargement of the liver and spleen, and profound anemia (1). Imaging examinations mainly show increased bone mineral density, thickened cortex, and medullary cavity occlusion. Characteristic radiological manifestations are the concentric circle sign of the ilium and the sandwich sign of the vertebral body (2). However, these radiological findings are not pathognomonic and can also be observed in other sclerosing bone disorders; definitive diagnosis requires combined clinical, radiological, and genetic evidence. The present study retrospectively reviewed the clinical records, genetic findings, and transplantation therapy outcomes of a pediatric patient with osteopetrosis. To our knowledge, this is the first report on a patient with compound heterozygous variants c.1567 + 2T>A and c.630C>G (p.Y210*), a genotype not previously described, with detailed post-HSCT longitudinal follow-up data. The results of the study may support early detection and intervention for this condition and enhance clinicians’ understanding and management of osteopetrosis.

## Case report

The proband was a Chinese girl who had shown abnormal bone development from early childhood. She was born at full-term following an uncomplicated pregnancy. At 36 months of age, she was 89 cm tall (third percentile), weighed 12 kg (10th percentile), and had a head circumference of 52 cm (>97th percentile). She also had enamel hypoplasia, only six erupted teeth, and a pigeon chest deformity. Developmental milestones were normal (independent sitting at six months, crawling at eight months, walking at 16 months) with unremarkable motor and intellectual development. She had a history of two fractures: left ankle (at 10 months old) and lower leg (at 3 years old), both of which were cured with plaster fixation. At the initial clinical visit to Children's Hospital Affiliated to Zhengzhou University on June 13, 2023, complete ophthalmologic and audiological evaluations were performed to screen for typical complications associated with osteopetrosis. Ophthalmic examination revealed mild stenosis of the optic foramen, but no optic nerve atrophy. Her visual acuity was within the normal range for her chronological age. Auditory brainstem response testing demonstrated normal bilateral hearing thresholds, with no conductive or sensorineural hearing impairments. Systematic abdominal physical examination showed no signs of hepatosplenomegaly. The liver was non-palpable, with an edge 0 mm below the right costal margin. The spleen was also non-palpable and positioned beneath the left costal margin. Abdominal ultrasonography completed concurrently with this visit provided quantitative visceral measurements: splenic parenchymal thickness was 16.3 mm, the main portal vein trunk diameter measured 5.7 mm, and the liver, gallbladder, pancreas, and bilateral kidneys all displayed normal size and morphology. Given these clinical features, a genetic disorder was strongly suspected, prompting additional sequencing analysis.

**Figure 1:**
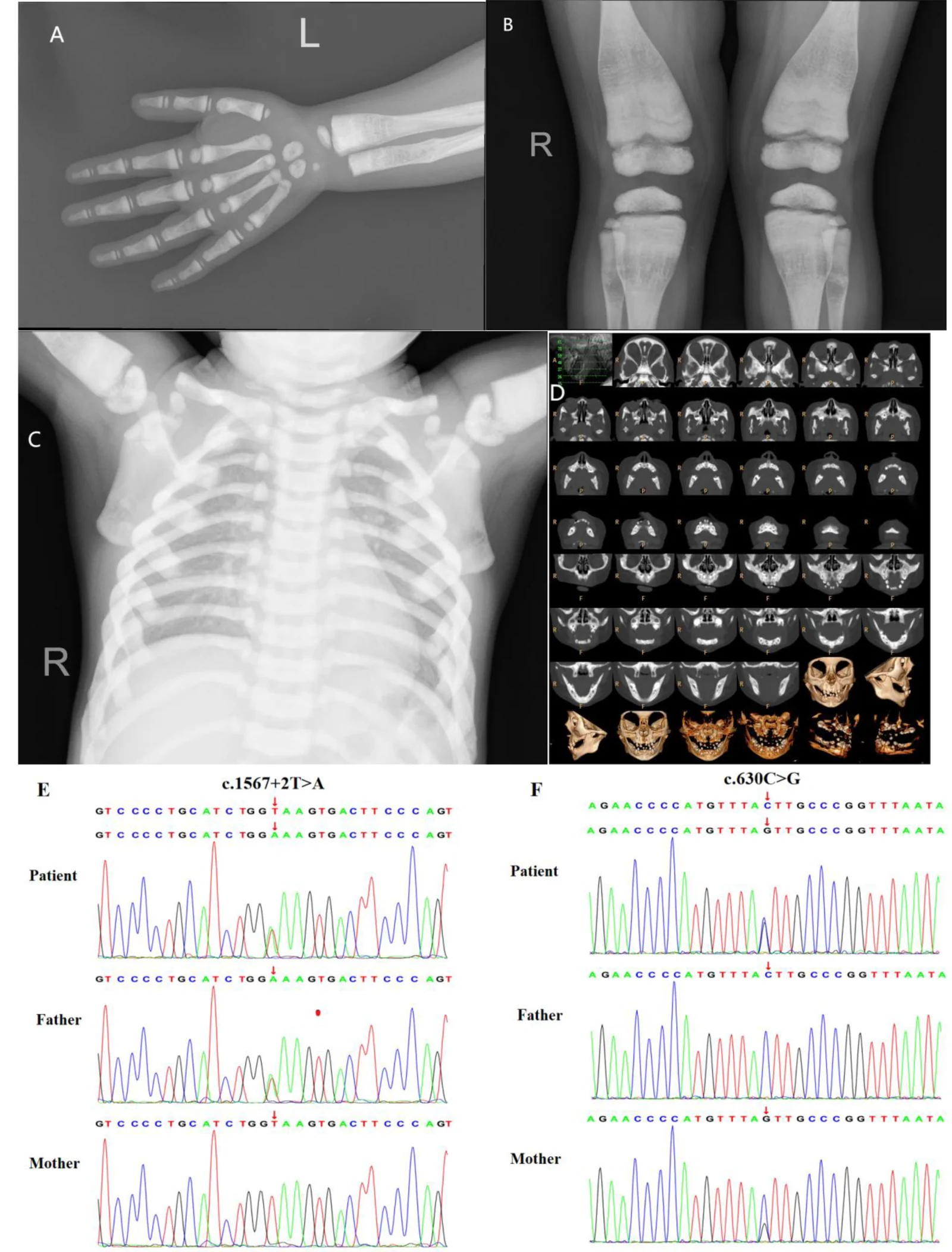
Anteroposterior x-ray of (**A**) the left wrist; (**B**) the knees; and (**C**) the chest. (**D**) Results of maxillofacial computed tomography + orthopedic 3D imaging. Variation sites of the *TNFRSF11A* gene (red arrows) inherited from the father (**E**) and the mother (**F**), detected by whole-exome sequencing.

Venous blood specimens (2 mL per individual) were obtained from the proband and both parents to perform whole-exome sequencing (WES). SIFT, PolyPhen2, and MutationTaster were used to predict variant effects on protein function, and pathogenicity was assessed following the standards recommended by the American College of Medical Genetics and Genomics. Routine blood tests, liver/kidney function, electrolytes, and cardiac color Doppler ultrasound were all unremarkable. The left wrist anteroposterior x-ray showed epiphyseal ossification of metacarpal bones, increased bone mineral density, and uneven/wrinkled cortical bone of the left ulna, suggesting osteopetrosis and old fractures (Figure 1A). Knee and chest anteroposterior x-rays demonstrated blurred bone texture, dense bone, a narrowed medullary cavity, and considerably increased density (Figures 1B, 1C). Maxillofacial computed tomography (CT) + orthopedic 3D imaging revealed dense bone, narrowed medullary cavity, significantly increased density, and abnormal maxillofacial tooth morphology (Figure 1D).

WES identified two heterozygous variants in the *TNFRSF11A* gene (NM_003839): 1) intron 9 splice site variant c.1567 + 2T>A (Sanger-confirmed, paternal inheritance; Figure 1E), classified as pathogenic (PVS1+PM2_supporting+PM3) – a classic splice site variant predicted to cause frameshift, nonsense-mediated mRNA decay (NMD) and loss of function (PVS1), absent from ExAC/gnomAD/dbSNP/1000Genomes (low-frequency; PM2_supporting), with pathogenic/suspected pathogenic variants in trans (PM3); 2) Exon 7 missense variant c.630C>G (p.Y210*, Sanger-confirmed, maternal inheritance; Figure 1F), classified as likely pathogenic (PVS1+PM2_supporting) – a nonsense variant predicted to induce NMD and loss of function (PVS1), with no prior reports in ExAC/gnomAD/dbSNP/1000Genomes (low-frequency; PM2_supporting).

The patient underwent HSCT at the Shanghai Children's Medical Center at the age of 3 years and 6 months, with her father as the donor (haploidentical match). At the moment of writing, at 4 years and 9 months, the child is in good general condition. Her height increased to 100 cm (12th percentile) and weight to 17.5 kg (22nd percentile), demonstrating evident catch-up growth relative to her pre-transplant anthropometric parameters. Her deciduous teeth have not yet fully erupted, and she has dental deformities, but no hepatosplenomegaly or anemia. The stenosis of the optic foramen is alleviated compared with the period before the transplant. The timeline of events and interventions is shown in Table 1.

**Table 1:** Timeline of the patient's medical history, diagnosis, intervention, and follow-up

Age	Event	Description
Birth	Uncomplicated full-term delivery	Uncomplicated pregnancy and delivery; no perinatal complications
10 months	First fracture	Left ankle fracture; treated with plaster fixation and healed
3 years	Second fracture	Lower leg fracture; treated with plaster fixation and healed
3 years (June 13, 2023)	Initial presentation	Referred to Children's Hospital Affiliated to Zhengzhou University; comprehensive ophthalmologic and audiological evaluations performed; radiologic examinations revealed diffuse osteosclerosis and narrowed medullary spaces
3 years	Genetic diagnosis	Whole-exome sequencing identified compound heterozygous variants in the *TNFRSF11A* gene: c.1567 + 2T>A (paternal) and c.630C>G, p.Y210* (maternal)
3 years 6 months	Hematopoietic stem cell transplantation (HSCT)	Underwent haploidentical HSCT from her father at Shanghai Children's Medical Center
4 years 9 months	Last follow-up	Height increased to 100 cm (12th percentile) and weight to 17.5 kg (22nd percentile); anemia resolved; optic foramen stenosis alleviated; no hepatosplenomegaly; no graft failure or major complications were observed

## Discussion

Osteopetrosis can be transmitted via several inheritance routes: autosomal recessive (ARO), autosomal dominant, and X-linked forms. Although the genetics of approximately 30% of human osteopetrosis cases remain uncharacterized, to date, at least 20 genes have been implicated in the disease, with *TCIRG1* being the most frequently affected. The proband in this study had osteopetrosis caused by a *TNFRSF11A* gene mutation. Sobacchi et al first characterized osteoclast-poor osteopetrosis caused by *RANKL* mutations (3), whereas Guerrini et al first reported on autosomal recessive osteopetrosis driven by biallelic *TNFRSF11A* (*RANK*) variants (4). Cumulative clinical and genetic studies have confirmed that biallelic pathogenic variants in *TNFRSF11A* cause type-7 autosomal recessive osteopetrosis (4,5). The missense variant identified in our patient alters an amino acid at a highly conserved evolutionary residue, which further supports the pathogenicity of this compound heterozygous genotype and the final diagnosis of *TNFRSF11A*-related ARO type 7. The *TNFRSF11A* gene, also known as *RANK*, encodes a 616-amino-acid receptor belonging to the tumor necrosis factor receptor superfamily; it is situated on chromosome 18 at position 18q21.33 (6). The *RANK* gene encodes the functional receptor for RANKL. The mutated residues seem to be highly conserved in evolution, so amino acid substitutions at these positions may change the folding of the extracellular domain, thereby modifying how the receptor engages with RANKL. *RANKL*, *RANK*, and related molecules influence osteoclast differentiation, leading to reduced osteoclast numbers; by contrast, mutations in other genes generally result in functional osteoclast impairment (7).

The diagnosis of osteopetrosis relies on typical clinical and radiological features, and/or on the detection of heterozygous disease-causing variants in *TNFRSF11A* via molecular genetic analysis. In early infancy, affected individuals often show macrocephaly, poor feeding, gradual vision loss, hearing impairment, bone marrow failure, marked anemia, and hepatosplenomegaly. Once the skull base nerve foramina become narrow or occluded, neurological compression symptoms and signs occur, the most common among which is damage to visual and auditory nerve function (8). Previously reported patients with *TNFRSF11A* mutations mostly had visual impairment, anemia, hepatosplenomegaly, and complete blindness (9).

At present, the diagnosis of osteopetrosis mainly relies on clinical manifestations/imaging examinations and genetic diagnosis. Most patients receive symptomatic and supportive treatment. Stem cell transplantation is currently an effective method for radical treatment of malignant infantile osteopetrosis, but it is expensive and risky (10). If the optic nerve has been damaged before transplantation, visual function cannot be restored. *RANK*-dependent ARO has been shown to benefit from HSCT, whereas forms caused by *RANKL* deficiency or OSTM1/CLCN7 defects generally respond poorly or not at all. This differential response can be explained by the cellular origin of the defect: *RANK* (*TNFRSF11A*) is expressed on hematopoietic osteoclast precursors, which are replaced by donor-derived cells after HSCT, restoring the RANK-RANKL signaling axis and normal osteoclast differentiation. In contrast, RANKL is primarily produced by mesenchymal cells and osteoblasts; thus, RANKL-deficient osteopetrosis is not corrected by hematopoietic stem cell replacement, as the defective microenvironment persists (11). The successful outcome in our patient therefore underscores the importance of precise genetic subtyping before transplantation, as it directly predicts the likelihood of HSCT efficacy. In the post-transplant period, patients are at increased risk for hypercalcemia, particularly in case of prolonged HSCT procedure (12). These observations expand the known clinical and molecular diversity of human ARO and underscore the importance of precise molecular diagnosis for guiding treatment decisions.
